# Caregiver Burden, Social Support, and Depressive Symptoms Among Caregivers of Patients With Heart Failure

**DOI:** 10.1097/jnr.0000000000000747

**Published:** 2026-05-11

**Authors:** Da-Young KIM, JiYeon CHOI, Youn-Jung SON

**Affiliations:** 1 Graduate School of Nursing, Chung-Ang University, Dongjak-gu, Seoul, South Korea; 2 Mo-Im Kim Nursing Research Institute, College of Nursing, Yonsei University, Seoul, South Korea; 3 Institute for Innovation in Digital Healthcare, Yonsei University, Seoul, South Korea; 4 Red Cross College of Nursing, Chung-Ang University, Seoul, South Korea

**Keywords:** caregiver burden, depressive symptoms, heart failure, mediation analysis, social support

## Abstract

**Background:**

Examining caregiver burden subgroups in family caregivers of patients with heart failure may help identify individuals at elevated risk. Also, although caregiver burden is known to be associated with social support and depressive symptoms, how these factors interact to produce depressive symptoms is unclear.

**Purpose:**

This study was designed to investigate the distinct subgroups of caregiver burden using latent profile analysis as well as the mediating role of social support in the relationship between caregiver burden profiles and depressive symptoms among heart failure family caregivers.

**Methods:**

Two hundred nine family caregivers (age ≥18 years) of older adults with heart failure were enrolled as participants. Caregiver burden, social support, and depressive symptoms were measured using self-administered questionnaires. Data were collected at the outpatient cardiology clinics of two university hospitals in Seoul, South Korea, between July 2022 and May 2023. Latent profile and mediation analyses were conducted using Mplus and PROCESS macro models.

**Results:**

The caregiver burden profiles were categorized into two groups: “lower burden” (78.9%) and “higher burden” (21.1%). The caregiver burden domain “emotional well-being” registered the highest scores in both lower and higher burden groups. Social support was shown to significantly mediate the relationship between higher caregiver burden (reference=lower caregiver burden group) and depressive symptoms.

**Conclusions:**

The findings indicate that promoting social support can buffer the negative impact of higher caregiver burden on depressive symptoms in family caregivers of older adults with heart failure. Health care professionals should develop interventions to strengthen social support to prevent depressive symptoms of family caregivers experiencing high caregiver burden.

## Introduction

Heart failure (HF) is a complex, progressive condition that affects 64 million patients worldwide, with high rates of readmission and mortality (Savarese et al., 2023). The incidence of HF increases with age, with an incidence of 20% in people aged 75 years and older (Díez-Villanueva et al., 2021). Older adults with HF often experience a combination of comorbidities, reduced physical function, and cognitive decline compounded by HF progression and the effects of normal aging (Díez-Villanueva et al., 2021). Consequently, older adults with HF struggle with self-care due in significant part to the complex related requirements, which often results in inadequate disease management (Savarese et al., 2023).

Family caregivers play a crucial role in managing symptoms, enhancing self-care, making decisions, and providing social support to patients with HF (Kitko et al., 2020). Particularly, family caregivers of older people with HF are generally more responsible for caring for their care recipients in all aspects of their lives, including assisting with more complex self-care or activities of daily living, than family caregivers of younger patients with HF (Schulz et al., 2020). The family caregiving burden may reduce the quality of care provided, leading to greater risks of readmissions and mortality for care recipients (Kitko et al., 2020) as well as higher burdens on family caregivers (Cheung et al., 2024). Greater understanding of family caregiver burdens is required to prevent negative outcomes and enhance the quality of life in HF family caregivers.

Caregiver burden refers to the multifaceted burden caregivers experience over time while caring for family members or loved ones (Lahoz et al., 2021; Liu et al., 2020). In a previous study, 86.7% of the HF family caregivers experienced a high level of caregiver burden (Hu et al., 2016). Moreover, caregiver burden is greater in older care recipients (Durante et al., 2019; Jackson et al., 2018). High levels of caregiver burden can lead to adverse outcomes, including psychological problems attributable to poor social support and depressive symptoms associated with reduced social activities, quitting work, and insufficient attentiveness to self-care (Suksatan et al., 2022).

Due to individual characteristics, family caregivers may experience varying degrees of caregiver burden in different dimensions (Choi et al., 2024). These characteristics can affect how stressors are appraised, available resources are accessed, and caregiving-related demands are managed, leading ultimately to varying levels of subjective burden (Lazarus & Folkman, 1984). Prior research has relied heavily on total caregiver burden scores to examine the factors of influence on caregiver burden and their effect on health outcomes in both patients and caregivers (Suksatan et al., 2022). This approach may overlook the multidimensional nature of caregiver burden and the possibility of subgroup effects based on individual burden profiles. Identifying these subgroups may inform the development of more effective, person-centered interventions to reduce burden among caregivers of patients with HF.

Latent profile analysis (LPA) is a categorical latent variable approach based on participant response patterns (Nylund et al., 2007) that may be used to identify individual specificities and reflect multidimensional interrelatedness (Mathew & Doorenbos, 2022). Consequently, LPA can effectively identify distinct subgroups in terms of caregiver burden of HF family caregivers, facilitating a more comprehensive understanding of each group’s unique characteristics.

Depressive symptoms are one of the most common adverse caregiving outcomes in HF family caregivers, affecting an estimated 6%–64% of those responsible for care provision (Lacerda et al., 2019). Depressive symptoms in family caregivers have been shown to negatively affect their quality of life (Clements et al., 2020). Moreover, depressive symptoms among caregivers can negatively influence self-care behavioral efficacy in patients with HF (Buck et al., 2015) and increase risks of unplanned readmission and mortality (Bidwell et al., 2017).

A significant association between caregiver burden and depressive symptoms was previously reported in family caregivers of HF patients (Suksatan et al., 2022). Also, a recent systematic review reported perceived caregiver burden as a significant risk factor for depressive symptoms in caregivers of older adults (Del-Pino-Casado et al., 2019). However, the mechanisms underlying these variables remain unclear. Therefore, exploring possible mediators of the link between caregiver burden and depressive symptoms in family caregivers of older adult patients with HF is crucial for designing appropriately individualized strategies to reduce the risk and severity of adverse caregiver health outcomes.

The negative emotional outcomes of caregiving are supported by the transactional model of stress and coping introduced by Lazarus and Folkman (1984). Under this model, individuals adapt by utilizing personal and environmental resources such as social support to perceive and appraise stressful situations and overcome them using coping behaviors. In one study, caregiver burden was found to relate to social support as a coping resource (Hu et al., 2016). In addition, several studies have shown lower social support to be linked to elevated symptoms of depression (Grant et al., 2020; Gutiérrez-Sánchez et al., 2023). Another recent study identified social support as a mediator in the relationship between caregiver burden and depressive symptoms in family caregivers of patients with HF (Graven et al., 2020). However, these and other related studies have been almost exclusively conducted in western countries, which limits generalizability to non-western countries and cultural settings. In South Korea, where family-centered caregiving is deeply rooted in Confucian values and shaped by strong social expectations (Fan et al., 2015), the experience of caregiving burden may differ significantly from that in western contexts. Therefore, examining caregiver burden in the South Korean context is essential for its unique caregiving dynamics and for developing culturally appropriate support systems.

A clearer understanding of the latent profiles of caregiver burden and the mediating role of social support in the relationship between caregiver burden profiles and depression in HF family caregivers is critical. In light of these issues, this study was designed to (1) determine the latent profiles of caregiver burden in the family caregivers of older patients with HF and (2) identify the mediating impact of social support on the relationship between the latent profiles of caregiver burden and depressive symptoms.

## Methods

### Study Design, Setting, and Participants

A cross-sectional design was adopted, and a convenience sample of family caregivers caring for older individuals with HF at outpatient cardiology clinics in two tertiary hospitals in Seoul, South Korea, was recruited. Data were collected from July 2022 to May 2023. Family caregivers were defined as those who provided most of the physical and emotional support for a patient. No legal relationship or cohabitation with the patient was required.

Inclusion criteria were: (1) family member of an older (≥65 years) care recipient with HF, (2) 18 years or older, (3) nonprofessional and unpaid, and (4) served in a caregiver role for at least six months. Exclusion criteria were: (1) suffering from a severe condition (e.g., terminal cancer requiring palliative care or end-stage renal disease requiring dialysis) and (2) diagnosis of a cognitive dysfunction (e.g., dementia) or psychiatric disorder (e.g., schizophrenia).

Of the 230 eligible family caregivers recruited, 16 declined to participate due to reasons such as unsuitable timing. After excluding five questionnaires due to missing answers, data from 209 participants were available for and used in subsequent analyses.

### Sample Size Calculation

The required sample sizes for LPA vary depending on factors such as total profile number and inter-profile distance (Morgan et al., 2016). Moreover, no definitive formula currently exists for determining the optimal sample size for LPA. Nylund-Gibson and Choi (2018) noted that, in simple models with well-separated classes, as few as 30 participants per profile may suffice. Tein et al. (2013) reported average entropy as higher at *N* = 250 than *N* = 1000 regardless of the number of latent profiles and that the statistical power of adjusted the Bayesian information criterion (BIC), sample-size adjusted BIC (aBIC), Lo-Mendell-Rubin (LMR), or bootstrap likelihood ratio test (BLRT) did not necessarily increase with sample size. In addition, Yang (2006) reported that ABIC showed good performance at *N* = 100 and 200. Therefore, while the sample size of 209 in this study may be considered sufficient for conducting LPA, some studies recommend larger sample sizes (300 ~ 500) as necessary to attain more robust results (Nylund et al., 2007; Tein et al., 2013). Given the relatively small sample size in this study, its findings should be interpreted with caution.

### Measures

#### General characteristics

The general characteristics data collected from the participants were: age, sex, educational level, marital status, working status, monthly income, body mass index (BMI) defined using the Korean society of obesity criteria (Haam et al., 2023), number of chronic diseases, care-recipient relationship type, living status with care recipients, duration of caregiving, and time spent on caregiving.

#### Caregiver burden

Caregiver burden was assessed in this study using the Korean version of the HF Caregiver Questionnaire (HF-CQ) developed by Strömberg et al. (2017). The construct and content validity of the Korean version of the HF-CQ have been previously confirmed (Strömberg et al., 2017). The tool comprises 21 items in three domains: physical well-being (5 items), emotional well-being (11 items), and lifestyle (5 items). Each item is evaluated on a 5-point Likert Scale (0–4), with reverse scoring applied to item 15. Total possible scale scores range from 0 to 100, with higher scores indicating greater caregiver burden. The Cronbach’s α for the original (English-language) version was .94 (Strömberg et al., 2017) and was .93 for the overall scale in this study.

#### Social support

Social support was assessed in this study using the Korean version (Jo et al., 2020) of the Enhancing Recovery in Coronary Heart Disease Social Support Instrument (ESSI) developed by Mitchell et al. (2003). The Korean version covers seven items distributed between the emotional and instrumental support (six items) and structural support (one item) dimensions. The emotional and instrumental support items are scored using a 5-point Likert Scale (1–5), while the structural support item (care recipient living/not living with their spouse) is scored using a 2 for no and 4 for yes. The total possible scale score ranges from 8 to 34, with higher scores indicating greater social support. The Cronbach’s α of the Korean version of the ESSI was .96 in a previous study and was .86 in this study.

#### Depressive symptoms

Depressive symptoms were assessed in this study using the Korean version of the original Patient Health Questionnaire-9 (PHQ-9; Spitzer et al., 1999) translated and published by Han et al. (2008). The PHQ-9 comprises nine items. The total possible scale score ranges from 0 to 27, with higher scores indicating more severe depressive symptoms. This scale has good reliability and validity in terms of identifying major depressive symptoms (Spitzer et al., 1999). The Cronbach’s α of the Korean version was .86 in Han et al. (2008) and was .77 in this study.

### Ethical Considerations and Data Collection Procedure

The study protocol was approved by the institutional research board of Chung-A University, Seoul, Korea (IRB No. 1041078-202205-HR-123). Before data collection, the participants were fully informed of the study purpose and procedures, guarantee of anonymity for participants, and the possibility of withdrawal. Those who voluntarily agreed to participate in the study provided written consent. All of the potential participants were introduced by one cardiologist who was part of the research team. Two trained research assistants explained the study objectives and procedures and obtained informed consent. The survey was conducted in a quiet room away from the care recipient and took ∼20 minutes to complete.

### Data Analysis

IBM SPSS Statistics version 28.0 (IBM Corp., Armonk, NY, USA) was utilized in data analysis. Descriptive statistics of frequency, percentage, and mean with *SD* were used to present participant baseline characteristics, while Pearson’s correlation coefficients were used to describe the associations among the main study variables.

Latent profiles of caregiver burden were identified using LPA run on Mplus version 8.8 software. The model fit indices, applied in accordance with previous studies (Nylund et al., 2007; Tein et al., 2013), were: the Akaike information criterion, BIC, aBIC, LMR, BLRT, entropy, and classification accuracy. Comparisons of the general characteristics as well as differences in caregiver burden, social support, and depressive symptoms based on caregiver burden profiles were analyzed using the χ^2^ test, independent sample *t* test, and one-way analysis of variance with a post hoc Duncan’s test. Predictors related to caregiver burden profiles were shown using binary logistic regression.

Finally, the mediating effect of social support on the association between LPA-based caregiver burden profiles and depressive symptoms was estimated using the PROCESS macro model 4 provided by Hayes (2022) in SPSS. PROCESS macro uses bootstrapping to estimate indirect effects, which are effective in controlling for type I errors. In this study, bootstrapping involving 10,000 resampling and 95% CIs was used to test the mediation models used to control for covariates, with the indirect effect deemed statistically significant when the 95% bootstrap CI did not include zero.

## Results

### General Characteristics of the Participants

In terms of participant general characteristics, the average age was 61.26 ± 13.26 years, 68.4% were women, and a majority were married (*n* = 178, 85.2%), unemployed (*n* = 119, 56.9%), and diagnosed with one or more chronic conditions (*n* = 119, 56.9%). Over half were the spouse of (58.9%) and lived together with (74.6%) the care recipient. The average duration of caregiving was 3.26 ± 3.89 years, and the average duration of care per week was 25.70 ± 22.94 hours (Table 1).

**Table 1:** Comparison of Caregiver Burden Profiles and Depressive Symptoms by Participant Baseline Characteristics (*N*=209)

Category	Total (*n*=209)	Lower Caregiver Burden (*n*=165)	Higher Caregiver Burden (*n*=44)	χ^2^ /*t* (*p*)	Depressive Symptoms (*n*=209)	*t* or *F* (*p*)
	*n* (%)	*n* (%)	*n* (%)		Mean±*SD*	
Age (years)				3.28		4.00
① <45	27 (12.9)	19 (11.5)	8 (18.2)	(.194)	3.00±2.83	(.020)
② 45-64	87 (41.6)	66 (40.0)	21 (47.7)		4.16±4.05	②>③
③ ≥65	95 (45.5)	80 (48.5)	15 (34.1)		2.76±2.91	
Sex				0.48		0.01
Male	66 (31.6)	54 (32.7)	12 (27.3)	(.489)	3.33±3.17	(.910)
Female	143 (68.4)	111 (67.3)	32 (72.7)		3.39±3.61	
Educational level				9.59		0.52
Below middle school	63 (30.1)	58 (35.2)	5 (11.4)	(.008)	3.00±2.79	(.594)
High school	72 (34.5)	54 (32.7)	18 (40.9)		3.56±3.14	
Above college	74 (35.4)	53 (32.1)	21 (47.7)		3.51±4.23	
Marital status				16.36		2.94
Married	178 (85.2)	149 (90.3)	29 (65.9)	(<.001)	3.08±3.29	(.004)
Unmarried	31 (14.8)	16 (9.7)	15 (34.1)		5.03±4.06	
Working status				1.93		0.07
Working	90 (43.1)	67 (40.6)	23 (52.3)	(.165)	3.30±3.21	(.792)
Not working	119 (56.9)	98 (59.4)	21 (47.7)		3.43±3.67	
Monthly incomes (10,000 Korean won)				1.20 (.274)		0.77 (.381)
<200	115 (55. 0)	94 (57.0)	21 (47.7)		3.18±3.26	
≥200	94 (45.0)	71 (43.0)	23 (52.3)		3.61±3.71	
BMI (kg/m^2^)				1.44		0.47
Underweight	10 (4.8)	8 (4.9)	2 (4.5)	(.697)	4.00±3.27	(.703)
Normal	75 (35.9)	56 (33.9)	19 (43.2)		3.29±4.11	
Overweight	53 (25.3)	44 (26.7)	9 (20.5)		3.00±2.84	
Obesity	71 (34.0)	57 (34.5)	14 (31.8)		3.65±3.21	
Number of chronic diseases				0.97 (.615)		1.36 (.260)
0	90 (43.1)	73 (44.2)	17 (38.6)		2.93±3.32	
1	59 (28.2)	44 (26.7)	15 (34.1)		3.58±3.33	
≥2	60 (28.7)	48 (29.1)	12 (27.3)		3.83±3.79	
Relationship with care recipient				7.41 (.006)		4.72 (.010)
Spouse	123 (58.9)	105 (63.6)	18 (40.9)		2.81±2.78	
Son or daughter	86 (41.1)	60 (36.4)	26 (59.1)		4.25±4.19	
Living with care recipient				0.52		0.31
Yes	156 (74.6)	125 (75.8)	31 (70.5)	(.473)	3.29±3.21	(.577)
No	53 (25.4)	40 (24.2)	13 (29.5)		3.60±4.17	
Duration of caregiving (years)				6.34 (.042)		0.82 (.441)
<1	67 (32.1)	46 (27.9)	21 (47.7)		3.82±4.30	
1–2	40 (19.1)	33 (20.0)	7 (15.9)		3.20±3.53	
≥3	102 (48.8)	86 (52.1)	16 (36.4)		3.15±2.78	
Time spent on caregiving (hours/week)				15.91 (.001)		1.40 (.243)
Q1 (≤10)	65 (31.1)	50 (30.3)	15 (34.1)		3.12±4.09	
Q2 (11–20)	69 (33.0)	53 (32.1)	16 (36.4)		3.65±3.44	
Q3 (21–40)	49 (23.4)	47 (28.5)	2 (4.5)		2.80±2.50	
Q4 (≥41)	26 (12.5)	15 (9.1)	11 (25.0)		4.35±3.36	

	Mean±*SD*	Mean±*SD*	Mean±*SD*	χ^2^ /*t*	*p*	
Caregiver burden	21.97±17.11	14.64±8.54	49.44±12.63	−17.25	<.001	
Physical well-being	15.62±20.03	7.88±10.85	44.66±19.96	−11.77	<.001	
Emotional well-being	25.42±18.41	18.04±10.40	53.10±15.28	−14.36	<.001	
Lifestyle	24.85±19.30	17.99±13.67	50.57±15.24	−13.71	<.001	
Social support	28.62±5.34	29.65±4.68	24.75±5.89	5.11	<.001	
Depressive symptoms	3.37±3.47	2.66±2.52	6.05±4.97	−4.37	<.001	

*Note.* BMI = body mass index; HF = heart failure; NYHA = New York Heart Association; Q = quartile.

In this study, the mean scores for caregiver burden, social support, and depressive symptoms were 21.97 (±17.11), 28.62 (±5.34), and 3.37 (±3.47), respectively. Strong correlations were observed among all three variables (all *p* <.001). In addition, in terms of the three domains of caregiver burden, the mean score for emotional well-being (25.42 ± 18.41) was highest, followed by lifestyle (24.85 ± 19.30) and physical well-being (15.62 ± 20.03).

In terms of depressive symptoms, statistically significant differences were found for age (*F* = 4.00, *p* = .020), marital status (*t* = 2.94, *p* = .004), and relationship with the care recipient (*t* = 4.72, *p* = .010). No significant differences were identified for the other general characteristics (Table 1).

### Identification of Latent Profile Analysis–Based Caregiver Burden Profiles

The results of the LPA of caregiver burden show entropy attained the highest classification accuracy (0.976) in the two-profile model, identifying the two-profile model as optimal based on the model fit indices (Akaike information criterion = 10918.481, BIC = 11122.363, aBIC = 10929.083, LMR = .038, and BLRT ≤.001).

**Figure 1:**
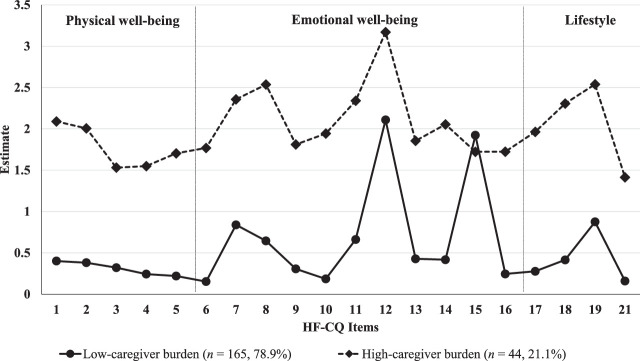
Patterns for Two Distinct Profiles of Caregiver Burden *Note.* HF-CQ = Heart Failure Caregiver Questionnaire.

The participants who reported relatively low response levels on the caregiver burden items were assigned to the “low-caregiver burden” group (*n* = 165, 78.9%), while those who reported high response levels were assigned to the “high-caregiver burden” group (*n* = 44, 21.1%). The estimated mean for each item using the two-profile model is shown in Figure 1.

The mean caregiver burden score was significantly lower in the lower burden group (*M* = 14.64, *SD* = 8.54) than the higher burden group (*M* = 49.44, *SD* = 12.63; *t* = −17.25, *p* < .001). Among the three burden domains, emotional well-being earned the highest scores in both groups, with means of 18.04 (*SD* = 10.40) in the lower burden group and 53.10 (*SD* = 15.28) in the higher burden group.

### Comparison of Caregiver Burden Profiles and Depressive Symptoms Based on Participant General Characteristics

When comparing participant characteristics across the caregiver burden profile groups, significant differences were observed in terms of educational level, marital status, relationship to the care recipient, duration of caregiving, and time spent on caregiving (Table 1). The higher burden group had significantly higher proportions of participants associated with a higher educational level (χ^2^ = 9.59, *p* = .008), unmarried status (χ^2^ = 16.36, *p* < .001), being a son or daughter (χ^2^ = 7.41, *p* = .006), having a longer duration of caregiving (χ^2^ = 6.34, *p* = .042), and spending more time on caregiving (≥41 hours per week; χ^2^ = 15.91, *p* = .001) compared with the lower burden group. However, no significant differences were found in terms of participant age, sex, working status, monthly income, BMI, number of chronic diseases, or living status with the care recipient.

The lower burden group reported significantly higher social support (*M* = 29.65, *SD* = 4.68) than the higher burden group (*M* = 24.75, *SD* = 5.89, *t* = 5.11, *p* < .001). Similarly, depressive symptoms were significantly lower in the lower burden group (*M* = 2.66, *SD* = 2.52) than the higher burden group (*M* = 6.05, *SD* = 4.97, *t* = −4.37, *p* < .001).

### Predictors Associated With the Caregiver Burden Profiles of Heart Failure Family Caregivers

Binary logistic regression analysis showed educational level, marital status, relationship with care recipient, duration of caregiving, and time spent in caregiving to be predictors of caregiver burden group (Table 2). Those with high caregiver burdens were more likely to have a higher educational level (adjusted odds ratio [*OR*] = 4.48, 95% CI: [1.26, 15.86]), be unmarried (adjusted *OR* = 3.49, 95% CI [1.23, 9.87]), have a shorter duration of caregiving (adjusted *OR* = 2.80, 95% CI [1.17, 6.73]), and spend more hours on caregiving per week (adjusted *OR* = 5.25, 95% CI [1.58, 17.50]) than those in the lower burden group.

**Table 2:** Factors Associated With Caregiver Burden Profiles (*N*=209)

Factor	Lower Caregiver Burden (ref) vs. Higher Caregiver Burden			
	Unadjusted *OR* [95% CI]	*p*	Adjusted *OR* [95% CI]	*p*
Educational level (ref. Below middle school)				
High school	3.87 [1.34, 11.14]	.012	4.18 [1.25, 13.99]	.020
Above college	4.60 [1.62, 13.06]	.004	4.48 [1.26, 15.86]	.020
Marital status (ref. Married)				
Unmarried	4.82 [2.15, 10.82]	<.001	3.49 [1.23, 9.87]	.019
Relationship with care recipient (ref. Spouse)				
Child	2.54 [1.28, 5.02]	.007	1.15 [0.40, 3.31]	.800
Duration of caregiving (ref. ≥3)				
<1	2.45 [1.17, 5.16]	.018	2.80 [1.17, 6.73]	.021
1-3	1.14 [0.43, 3.02]	.792	1.38 [0.46, 4.13]	.570
Time spent in caregiving (ref. Q1 (≤10))				
Q2 (11-20)	1.01 [0.45, 2.25]	.988	1.78 [0.63, 5.02]	.275
Q3 (21-40)	0.14 [0.03, 0.65]	.012	0.21 [0.04, 1.08]	.062
Q4 (≥41)	2.44 [0.93, 6.44]	.071	5.25 [1.58, 17.50]	.007

*Note.* Adjusted for age, marital status, and relationship with care recipient; Low-caregiver burden is used as the reference.

### Mediation Analysis of Social Support on the Association of Caregiver Burden Profiles With Depressive Symptoms

**Figure 2:**
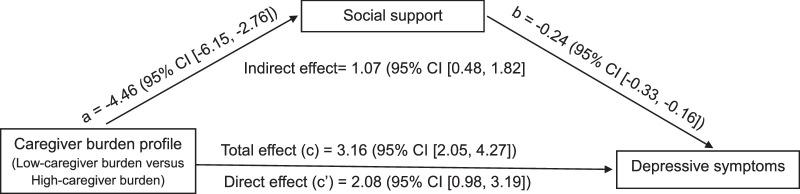
The Mediating Effect of Social Support on the Relationship Between Caregiver Burden Profiles and Depressive Symptoms of Family Caregivers *Note.* Low-caregiver Burden Is Used as the Reference. Adjusted for Age, Marital Status, and Relationship With Care Recipients. a: The Relationship of Caregiver Burden and Social Support; b: The Relationship of Social Support and Depressive Symptoms; c: The Relationship of Caregiver Burden and Depressive Symptoms; c’: The Relationship of Caregiver Burden and Depressive Symptoms After Adopting Social Support as a Mediator.

In Figure 2 and Table 3, the results are shown adjusted for age, marital status, and caregiver-care recipient relationship as covariates, with lower caregiver burden used as the reference. The results show support was significantly lower in the higher burden group (*B* = −4.46, 95% CI [−6.15, −2.76]) than the lower burden group, and that lower social support was associated with worse depressive symptoms (*B =* −0.24, 95% CI [−0.33, −0.16]). The total effect of caregiver burden on depressive symptoms was significant, with the higher burden group reporting average symptom scores that were 3.16 lower than the lower burden group (95% CI [2.05, 4.27]). After controlling for social support, the direct effect remained significant (95% CI [0.98, 3.19]), and the indirect effect through social support was also significant (*B* = 1.07, 95% CI [0.48, 1.82]). This result supports that social support significantly mediates the relationship between caregiver burden and depressive symptoms.

**Table 3:** The Mediating Effect of Social Support on the Relationship Between Caregiver Burden Profile and Depressive Symptoms (*N*=209)

Path	Model Pathways	*Β*	*SE*	β	*t*	95% CI	
						LLCI	ULCI
Path a	High-caregiver burden → Social support	−4.46	0.86	−0.84	−5.19	−6.15	−2.76
Path b	Social support → Depressive symptoms	−0.24	0.04	−0.37	−5.63	−0.33	−0.16
Path c’	Direct effect: High-caregiver burden → Depressive symptoms	2.08	0.56	0.60	3.73	0.98	3.19
Path c	Total effect: High-caregiver burden → Social support → Depressive symptoms	3.16	0.56	0.91	5.60	2.05	4.27
	Indirect effect: High-caregiver burden → Social support → Depressive symptoms	1.07	0.35			0.48	1.82

*Note.* Caregivers’ age, marital status, and relationship with care recipient are controlled statistically. Low-caregiver burden is used as the reference. LLCI = lower limit confidence interval; ULCI = Upper limit CI.

## Discussion

The main findings of this study revealed the latent profiles of two distinct caregiver burden profiles, namely lower burden (78.9%) and higher burden (21.1%). In both groups, the highest average score was for emotional well-being. In the study sample, social support significantly mediated the association between caregiver burden profile and depressive symptoms after adjusting for covariates. While few previous related studies have compared their findings directly with those of other studies, Graven et al. (2023) identified three classes of caregivers, namely with adequate resources (42.3%), at risk of decompensation (25.1%), and with inadequate resources (32.6%), based on indicators of coping resources and stress in caregivers of individuals with HF. The findings of this study are partly supported by the findings of Graven et al. (2023) in terms of supporting an association between caregivers with few coping resources and higher level of caregiving burden.

Interestingly, emotional well-being, as measured using the HF-CQ (Strömberg et al. 2017), recorded the highest average scores in both caregiver burden groups. Also, the results further demonstrate that caregiver burden is significantly related to depressive symptoms. Research using the same measure to study Chinese caregivers reported that caring for individuals with HF significantly affects the emotional well-being of caregivers, with a notable psychological impact (Jackson et al., 2018). A systematic review by Suksatan et al. (2022) reported that the role of HF caregiving significantly impacts the physical, psychological, and social dimensions of caregivers’ lives. Conversely, a study conducted on caregivers of European HF patients found the emotional dimension of caregiver burden to earn the lowest mean scores (Durante et al., 2023). Therefore, interpretations of the results in this study should be made with caution, as different populations and assessment tools can yield varying results. Notably, item 15 of the emotional well-being dimension, which is used to assess the degree of emotional support, was an exception, with the lower burden group reporting lower levels of support than their higher caregiving burden peers. This finding may be interpreted as the lower burden group experiencing fewer caregiving-related emotional difficulties and thus subjectively perceiving less need for emotional support from family or friends. A significant association was identified in this study between caregiver burden and depressive symptoms among family caregivers, with higher levels of caregiver burden positively correlated with greater depressive symptoms, which is consistent with the findings of Suksatan et al. (2022). Importantly, these results demonstrate that the association between high caregiver burden and a higher depressive symptoms score is mediated by social support, using lower burden as the reference point. This finding in this study is consistent with existing evidence (Graven et al., 2023; Suksatan et al., 2022) and supports that social support can buffer the negative influence of caregiver burden on depressive symptoms, suggesting social support may buffer the negative influence of caregiver burden on depressive symptoms in line with stress and coping theory (Lazarus & Folkman, 1984). According to this theory, in the stressful context of caregiving, the coping resource of social support can mitigate the emotional consequences of depression.

In this study, the average duration of family caregiving was greater than three years, and the amount of caregiving provided was ∼26 hours per week. These numbers are higher than those reported by Suksatan et al. (2022), whose participants had provided care for one year or more and spent an average of over four hours per day caring for patients with HF. Suksatan et al. emphasized that longer caregiving time is associated with a higher risk of caregiver burden, which can result in worse depressive symptoms. Accordingly, social support interventions should be targeted at those with high burdens of care, determined in light of their overall caregiving duration and hours of caregiving provided per day. Moreover, to inform the development of social support interventions aimed at reducing burden among family caregivers of patients with HF, health care professionals should evaluate the extent and duration of caregiving needed to support patients’ self-care behaviors and activities of daily living throughout the illness trajectory.

The sample used in this study had a mean age of over 60 years, and the majority of participants were female spouse caregivers who cohabited with their care recipients. Also, more than half of the participants had multiple chronic diseases. Confucian family values place strong emphasis on family bonds and responsibility, viewing the family as having ontological priority over individual members (Fan et al., 2015). In this cultural context, spouses and children are perceived as the primary caregivers within the family unit and are naturally expected to provide care (Fan et al., 2015). Despite its rapid industrialization and economic development, traditional familial values in South Korea remain strongly entrenched. Therefore, formal support from the community or national organizations may be needed to reduce the burden on family caregivers (Kitko et al., 2020). Interventions that include social support components such as peer support, regular home visits, and consultations with health care professionals may be beneficial in lowering caregiver burden and preventing or managing depressive symptoms in the family caregivers of patients with HF (Lauritzen et al., 2015; Hu et al., 2016).

### Strengths and Limitations

The main findings of this study highlight the importance of assessing caregiver burden profiles early in the family caregivers of individuals with HF. The findings may contribute to the development of personalized social support interventions to reduce the adverse impact of caregiver burden on depressive symptoms in this vulnerable group.

Several limitations of this study should be considered. First, the cross-sectional design with convenience sampling approach used prevents the establishment of causality between the main variables. Second, the disease-related characteristics of the care recipients, for example, HF severity, time since diagnosis, HF treatments, medication and pacemaker use, physical functioning, and activity levels, were not considered in this study, although they may also affect caregiver burden. Furthermore, family network, co-caregivers, intensity of caregiving, and caregiving styles were not considered as caregiver characteristics. Third, the sample size in this study was the minimum size required for analysis. Larger sample sizes (e.g., over 300 participants) should be used in future studies to better identify distinct caregiver burden groups. Finally, the use of a self-administered questionnaire format in this study increased the risks of social desirability response bias and data misreporting.

### Future Research

To validate the findings and extend their generalizability, longitudinal or intervention studies targeting various cultural backgrounds and multicenter settings should be conducted. More studies are required to identify caregiver burden profiles over time that include consideration of the types of caregiving approach used by family caregivers vs. paid / formal caregivers. Finally, future studies should collect more detailed information on the characteristics of care recipients and family caregiving.

### Conclusions

The findings of this study support distinguishing the family caregivers of older individuals with HF into the two distinct profile categories of “lower” and “higher” caregiver burden. Using lower caregiver burden as the reference group, social support was shown to be a significant mediator in the association between caregiver burden and depressive symptoms. Therefore, health care professionals should consider arranging, in collaboration with community and national institutions, the formal/informal support necessary to help reduce caregiver burden in family caregivers of individuals with HF. Moreover, caregiving duration and time spent on caregiving per day should be considered in developing interventions to improve the relevance and accuracy of support provided.
